# Beliefs about COVID-19 as a threat to values are related to preventive behaviors and fear of COVID-19

**DOI:** 10.1177/13591053221142348

**Published:** 2023-01-02

**Authors:** Marina Iosifyan, Galina Arina, Valentina Nikolaeva

**Affiliations:** 1 University of St Andrews, Scotland; 2 Lomonosov Moscow State University, Russia

**Keywords:** COVID-19, fear, health, health behaviors, values

## Abstract

We investigated factors related to preventive behaviors and fear of COVID-19: values and beliefs about threat to values because of COVID-19. In two studies, participants reported their own values and evaluated how COVID-19 may threaten values. They also reported their preventive behaviors (washing hands, wearing a facial mask, keeping social distance, and avoiding public places) and fear of COVID-19. COVID-19 is perceived as a threat to personal focused values (openness and self-enhancement values) rather than social focused values (conservation and self-transcendence values). Both value importance and perceived threats to values are related to preventive behaviors and fear of COVID-19. Greater importance of conservation values was related to engaging in preventive behaviors and increased fear of COVID-19. Perceived threats to personal focused values (self-enhancement and openness values) were also related to engaging in preventive behaviors and fear of COVID-19.

## Introduction

Coronavirus Disease (COVID-19) is an infectious disease, which spread across the globe in 2020. Scientists from different countries reported that the COVID-19 pandemic has contributed to a major mental health burden, including increased levels of stress, general anxiety, depressive symptoms, and fear of infection (Huang and Zhao, 2020; Mertens et al., 2020; Park et al., 2020; Pieh et al., 2021). Factors related to these symptoms can be targets for psychological interventions which aim to improve mental health.

**Figure 1.**
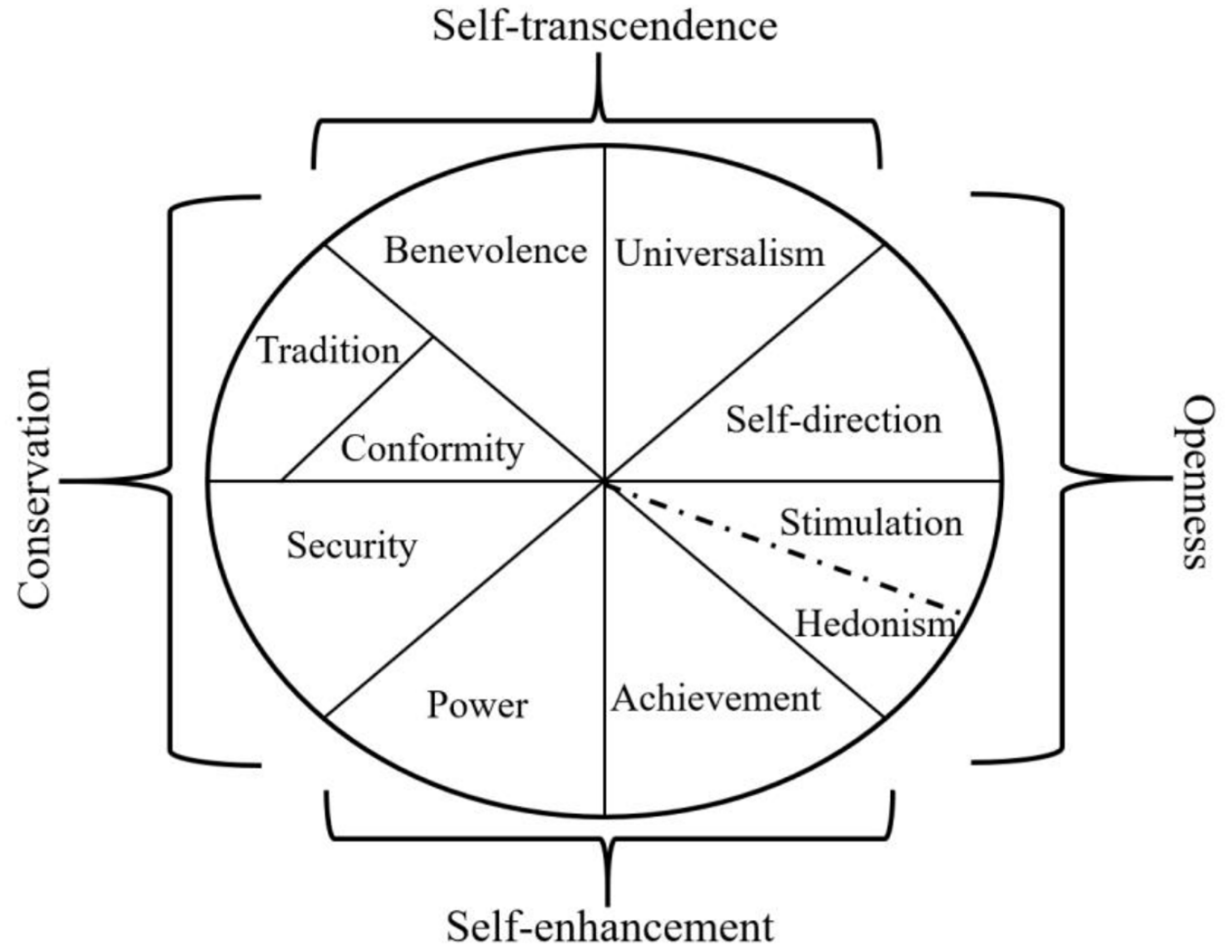
The circumplex model of values (Schwartz, 1992).

One important variable that has been subject to research in the context of COVID-19 is the concept of values (Wolf et al., 2020). Values are abstract motivations which predict attitudes and behaviors (Schwartz, 2003). In line with Schwartz’s model of values, there are four higher-order values: openness (independent thought and action–choosing, readiness for change), self-enhancement (pursuit of one’s own interests and success), self-transcendence (concern for the interests of others), and conservation (order and resistance, see Figure 1).

Since values are related to emotions (Nelissen et al., 2007) they may partly explain fear of COVID-19. In line with Schwartz’s theory of values, worries (a subtype of anxiety) are related to value priorities (Schwartz et al., 2000). People who attach particular importance to *personal focused values* (self-enhancement and openness values) experience higher level of *micro worries* (worries about the self), whereas people who attach particular importance to *social focused values* (self-transcendence and conservation values) experience higher level of *macro worries* (worries about the society or the world; Schwartz et al., 2000). This theory was applied to explain how values are related not only to worries, but to fear. For instance, it was applied to understand how values are related to the fear of crime as a macro worry (a threat to self; Barni et al., 2016; Russo and Roccato, 2009). Moreover, earlier research showed that perceived threats to personal focused values are related to higher fear of health impairments (e.g. blindness, motor impairments, cognitive impairments; Iosifyan and Arina, 2021). In case of COVID-19, however, these relations between values and worries can be different. Indeed, COVID-19 is a personal health threat—that is, a threat to personal focused values. However, COVID-19 can also represent a threat to social focused values because it has a major impact not only on one’s personal health, but also the health of others, particularly of vulnerable social groups (e.g. people with chronic diseases, elderly people), quality of life, economy, and cultural life of the society. Thus, we might expect that both personal and social focused values will be associated with fear of COVID-19.

A second line of reasoning on links between values and fear of COVID-19 is associated with another criteria of value organization: relations to anxiety (Schwartz, 2016). Some values are *anxiety-based* (values expressing the need of self-protection: conservation and self-enhancement values), while others—*anxiety-free* (values expressing the need of self-expansion: openness and self-transcendence values; Schwartz, 2016). Thus, anxiety-based values can be related to fear of COVID-19. A recent longitudinal study investigated personal values during the COVID-19 pandemic and found that conservation and self-enhancement values (anxiety-based values) increased during the pandemic, while self-transcendence and openness values (anxiety-free) decreased (Daniel et al., 2022). A recent cross-sectional study also found that security values (conservation values) and power values (self-enhancement values) were strongly related to COVID-19 worries (Fisher et al., 2021). Thus, we might expect that anxiety-based conservation and self-enhancement values will be positively related to fear of COVID-19.

Indeed, fear of COVID-19 can be related to individuals’ own value priorities (social vs personal focused, anxiety-based vs anxiety-free), but also to beliefs about *value threat*. For example, individuals who highly value conservation might fear COVID-19, if they believe that COVID-19 is a threat to conservation values. On the other hand, if they don’t believe that COVID-19 is a threat to conservation values, they might not experience fear, even if they highly value conservation. For this reason, the present study investigates both individuals’ value priorities and perceived value threats.

Fear of COVID-19 plays a motivational role and increases preventive behaviors. Previous research found that fear of COVID-19 is positively related to adherence to lockdown rules (Winter et al., 2020). Values associated with fear of COVID-19 can be also associated with preventive behaviors. Earlier research showed that values express themselves in certain behaviors. For example, self-transcendence values are related to environmental behaviors (Milfont et al., 2010). Values can play an important role in guiding preventive behaviors during COVID-19 pandemic (Wolf et al., 2020). Understanding links between values, perceived value threats, and preventive behaviors is important, because if values are related to them, they can be used to stimulate engagement in these behaviors. For example, developing messages appealing to certain values (e.g. self-transcendence values: engaging in preventive behaviors in order to protect others) and thus increasing persuasiveness of these messages.

As far as we are aware, the present research is the first to investigate the role of own’s value priorities and perceived value threats in fear of COVID-19, as well as in preventive behaviors (e.g. keeping social distance). In two studies, conducted during COVID-19 pandemic in 2020 we investigated relations among values, fear, and preventive behaviors. We expected that the importance of anxiety-based conservation and self-enhancement values, as well as perceived threat to these values, would be positively related to fear of COVID-19 and preventive behaviors.

## Method

### Participants

Power analysis conducted in G*Power (Faul et al., 2007) indicated that at least 109 participants are required to test a regression model with a medium effect size with eight predictors, alpha = 0.05 and power = 0.80. In Study 1, 120 participants were recruited in March 2020 during the COVID-19 pandemic lockdown. Ten participants failed the attention check and were excluded from further analysis.^1^ Final sample included 110 participants from 19 to 68 years (*M* = 38.83, SD = 10.98, 49 males, 59 females, 1 non-binary, 1 did not report their gender). In Study 2, 140 participants were recruited in September 2020 when lockdown restrictions were lifted. Eight participants failed the attention check and were excluded from further analysis. One hundred and thirty-two participants from 18 to 68 years were included in the analysis (*M* = 35.26, SD = 11.37, 78 males, 54 females). All participants were recruited on a platform for online research (Toloka.ai), lived in Russia and received compensation for their participation.

### Fear of COVID-19

Participants completed the questionnaire developed by Mertens et al. (2020) which measured fear of COVID-19. It includes eight items (e.g. “*I am very worried about the COVID-19*”) evaluated on a scale from 1 (strongly disagree) to 5 (strongly agree). It was back translated in Russian language. The internal consistency of this scale in present study was high (Cronbach’s alpha = 0.85 in Study 1 and 0.83 in Study 2).

### Values

Participants completed the Short Schwartz’s Value Survey (SSVS; Lindeman and Verkasalo, 2005). The ten values were collapsed into four higher order values: openness (self-direction, stimulation, hedonism; Cronbach’s alpha = 0.54 in Study 1 and 0.79 in Study 2), self-enhancement (achievement, power, *r*(110) = 0.415, *p* < 0.0001 in Study 1, *r*(131) = 0.340, *p* < 0.0001 in Study 2), self-transcendence (universalism, benevolence, *r*(110) = 0.500, *p* < 0.001 in Study 1, *r*(132) = 0.584, *p* < 0.001 in Study 2), and conservation (conformity, tradition, security, Cronbach’s alpha = 0.77 in Study 1 and 0.76 in Study 2). The data indicated an excellent fit to the hypothesized structure (Tucker’s Coefficient of Congruence = 0.995 in Study 1 and 0.986 in Study 2).

### Perceived threats to values

Participants completed the Short Schwartz’s Value Survey to measure perceived value threat. They were asked to evaluate how values could be threatened because of the COVID-19 (“*Imagine a person who has a COVID-19. As per your view, what values can be threatened as a result of his/her current health state*?”) on a scale from 0 (definitely will not be threatened) to 5 (definitely will be threatened). The ten values were collapsed into four higher order values as described above: openness (Cronbach’s alpha = 0.80 in Study 1 and 0.78 in Study 2), self-enhancement (*r*(110) = 0.470, *p* < 0.0001 in Study 1, *r*(132) = 0.477, *p* < 0.0001 in Study 2), self-transcendence (*r*(110) = 0.522, *p* < 0.001 in Study 1, *r*(132) = 0.589, *p* < 0.001 in Study 2), and conservation (Cronbach’s alpha = 0.77 in Study 1 and 0.81 in Study 2). The data indicated an excellent fit to the hypothesized structure (Tucker’s Coefficient of Congruence = 0.994 in Study 1 and 0.988 in Study 2).

### Preventive behaviors

Participants reported how often they wash their hands, wear a facial mask, maintain social distance, and avoid public places (1 = never, 5 = always). These four items were collapsed in one index of preventive behaviors (Cronbach’s alpha = 0.77), with a higher score indicating greater engagement in preventive behaviors.

### Procedure

After providing informed consent, participants first reported their fear of COVID-19 (Studies 1–2), preventive behaviors (Study 2). They next reported their own values, and perceived threats to values (Studies 1–2).

## Results

Firstly, we investigated perceived threats to values, aggregating data across studies 1–2 (for descriptive statistics, see Table 1). A repeated measures ANOVA with the Greenhouse-Geisser correction indicated that perceived threats to self-transcendence, self-enhancement, openness, and conservation values varied significantly, *F*(2.45, 589.97) = 72.17, *p* < 0.001, η_p_^2^ = 0.230.^2^ Paired sample t-tests with Bonferroni’s multiple testing correction showed that openness values were threatened more than conservation values, *t*(241) = 10.01, *p* < 0.0001, *d* = 0.64. Self-enhancement values were threatened more than self-transcendence values, *t*(241) = −5.14, *p* < 0.001, *d* = 0.33.

**Table 1.** Means, standard deviations, and correlations of all variables.

	*M*	SD	Correlations (*r*)								
			1.	2.	3.	4.	5.	6.	7.	8.	9.
Own values											
1. ST	3.25	1.54	1								
2. SE	2.36	1.36	0.245**	1							
3. OP	3.27	1.20	0.380**	0.421**	1						
4. CO	3.27	1.35	0.545**	0.347**	0.364**	1					
Perceived value threats											
5. ST	1.84	1.50	0.099	0.242**	0.124	0.247**	1				
6. SE	2.34	1.44	0.185**	0.218**	0.209**	0.179**	0.467**	1			
7. OP	3.16	1.40	0.163**	0.222**	0.304**	0.250**	0.322**	0.447**	1		
8. CO	2.09	1.50	−0.002	0.231**	0.120	0.207**	0.766**	0.513**	0.343**	1	
Dependent variables											
9. Fear of COVID-19	25.67	7.11	0.144*	0.135**	0.106	0.288**	0.254**	0.335**	0.289**	0.222**	1
10. Behaviors	3.73	0.90	0.290**	0.129	0.040	0.365**	0.155	0.242**	0.257**	0.154	0.685**

ST: self-transcendence values; SE: self-enhancement values; OP: openness to change values; CO: conservation values.

* *p* < 0.05. **p < 0.01.

Secondly, fear of COVID-19 was regressed onto the main effects of value importance ratings and perceived value threats; the data was aggregated across both studies (see Table 2, Model 1). Regarding importance, we found that conservation values were positively related to fear of COVID-19, indicating that individuals who attach greater value to conservation express more fear of COVID-19, *b* = 1.26, SE = 0.40, β = 0.24, *p* = 0.002. Regarding threat, we found that perceived threat to self-enhancement values was positively related to fear of COVID-19, indicating that individuals who believe that COVID-19 threatens self-enhancement values express more fear of COVID-19, *b* = 1.15, SE = 0.38, β = 0.23, *p* = 0.003.

**Table 2.** Regression models testing the effects of values on dependent variables across studies.

	Model 1				Model 2			
	Fear of COVID-19				Behaviors			
	*B*	*SE*	β	*p*-Value	*b*	*SE*	β	*p*-Value
Value importance								
ST	−0.18	0.37	−0.04	0.627	0.07	0.07	0.12	0.318
SE	−0.04	0.36	−0.01	0.903	0.02	0.06	0.03	0.740
OP	−0.34	0.42	−0.06	0.420	−0.20	0.07	−0.29	0.006
CO	1.26	0.40	0.24	0.002	0.21	0.08	0.32	0.014
Perceived threat to values								
ST	0.49	0.45	0.10	0.282	−0.01	0.08	−0.02	0.884
SE	1.15	0.38	0.23	0.003	0.07	0.07	0.10	0.322
OP	0.68	0.36	0.13	0.063	0.17	0.06	0.26	0.011
CO	−0.31	0.47	−0.07	0.508	−0.05	0.09	−0.08	0.589
Model’s *R*^2^	0.15				0.17			

Variance inflation factors of the independent variables were analyzed and no multicollinearity issues were detected (VIF < 4).

ST: self-transcendence values; SE: self-enhancement values; OP: openness values; CO: conservation values.

Preventive behaviors were regressed onto the main effects of value importance ratings and perceived value threats (see Table 2, Model 2). Regarding importance, we found that importance of openness values was negatively related to preventive behaviors, while conservation values were positively related to them, *b* = −0.20, SE = 0.07, β = −0.29, *p* = 0.006; *b* = 0.21, SE = 0.08, β = 0.32, *p* = 0.014. Regarding threat, perceived threat to openness values was positively related to behaviors, *b* = 0.17, SE = 0.06, β = 0.26, *p* = 0.011. That is, individuals who believe that COVID-19 threatens openness values engage in preventive behaviors more.

## Discussion

This research investigated relations among value importance, perceived value threats, fear of COVID-19, and preventive behaviors. The fact that COVID-19 was perceived as a threat to self-enhancement rather than to self-transcendence values, and openness rather than to conservation values, indicates that personal focused values are threatened more than social focused values. This finding is consistent with reasoning that health threats are perceived as threats to the self rather than to the society. This indicates that despite COVID-19 threatening the health of others, it is still perceived as a major threat to self rather than to others. This might explain why longitudinal studies found that self-transcendence values decreased, while self-enhancement increased during the pandemics: the former are perceived as threatened less compared to the latter (Daniel et al., 2022). This finding also shows that COVID-19 as a threat is perceived differently compared to ecological threats, since ecological threats are positively related to social focused values: self-transcendence, and negatively—to personal focused values of self-enhancement (Schultz et al., 2005).

Interestingly, we found that openness values are threatened more than conservation values, which involve security values. That is, individuals believe that COVID-19 threatens goals related to independence, freedom, and stimulation to a greater extent than goals related to security and safety. This finding can be linked to the observation that some people do not necessarily perceive COVID-19 as a life threat, but rather as a threat to their lifestyles and quality of life. Indeed, COVID-19 may represent a life threat, because it can be life-threatening, but at the same time, it can be also perceived as a quality-of-life threat, because it has generated many major lifestyle restrictions (e.g. restrictions on traveling, socializing, business and leisure activities, and financial constrains) and is not life-threatening for some groups of people. It is thus possible that participants who perceive COVID-19 as a life threat, believe that it threatens conservation values more, while those who perceive it as a quality-of-life threat—believe that it threatens openness values more. Future studies may address this question asking participants to indicate their perception of COVID-19 as a life threat or a quality-of-life threat.

The second major finding of this research is that perceived value threats are related to fear of COVID-19. Perceived threat to self-enhancement values was positively related to fear of COVID-19. Self-enhancement values are both anxiety-based and personal focused. The fact that threat to these values is positively related to negative symptoms during COVID-19 pandemics is in line with Schwartz’s et al. (2000) assumptions on values and worries. This finding may indicate that policies and messages on alternative ways of fulfilling self-enhancement values may decrease fear of COVID-19. Alternatively, including threat to self-enhancement values in fear appeal messages may motivate people to engage in preventive behaviors. Future studies may test these effects of threat to self-enhancement values.

A third major finding of this research is that values are associated with preventive behaviors. Perceived threat to personal focused values of openness is positively related to preventive behaviors (e.g. wearing masks and social distancing). Interestingly, individuals’ importance attached to openness values were negatively related to these behaviors, whereas conservation values were positively associated with the behaviors. This implies that people who value openness more (self-direction, stimulation, and excitement) and conservation less (security and safety) engage in preventive behaviors less, but people who perceive COVID-19 as a threat to these values—engage in preventive behaviors more. This finding suggests that different mechanisms can underly the effects of own values and perceived value threats. Moreover, this finding might have some practical implications. Preventive behaviors can be positioned as measures to protect one’s openness values now or in the future, which might increase engagement in these behaviors.

A primary main limitation of Studies1–2 is their correlational design. It is not clear whether perceived threat to values increases the fear of COVID-19, or fear of COVID-19 increases perceived threat to values. This relation can be bidirectional: perception of threats can increase worries and anxiety, but anxiety in its turn can increase overestimation of risks and threats (Lerner and Keltner, 2001; Shechner et al., 2012).

Overall, it was found that COVID-19 threatens personal focused values (openness to change and self-enhancement values) more compared to social focused ones (conservation and self-transcendence values). Perceived threats to personal focused self-enhancement and openness to change values are positively related to fear of COVID-19 and preventive behaviors.

## Supplemental Material

sj-pdf-1-hpq-10.1177_13591053221142348 – Supplemental material for Beliefs about COVID-19 as a threat to values are related to preventive behaviors and fear of COVID-19 Click here for additional data file. Supplemental material, sj-pdf-1-hpq-10.1177_13591053221142348 for Beliefs about COVID-19 as a threat to values are related to preventive behaviors and fear of COVID-19 by Marina Iosifyan, Galina Arina and Valentina Nikolaeva in Journal of Health Psychology

## Notes

1. The attention check involved asking the participants to select a certain response option (e.g. “Please select the answer 1”).

2. All analysis was conducted in IBM SPSS 26

## References

[bibr1-13591053221142348] Barni D Vieno A Roccato M , et al. (2016) Basic personal values, the country’s crime rate and the fear of crime. Social Indicators Research 129: 1057–1074.

[bibr2-13591053221142348] Daniel E Bardi A Fischer R , et al. (2022) Changes in personal values in pandemic times. Social Psychological and Personality Science 13: 572–582.

[bibr3-13591053221142348] Faul F Erdfelder E Lang AG , et al. (2007) G*Power 3: A flexible statistical power analysis program for the social, behavioral, and biomedical sciences. Behavior Research Methods 39(2): 175–191. 17695343 10.3758/bf03193146

[bibr4-13591053221142348] Fisher A Roberts A McKinlay AR , et al. (2021) The impact of the COVID-19 pandemic on mental health and well-being of people living with a long-term physical health condition: A qualitative study. BMC Public Health 21: 1801. 34620136 10.1186/s12889-021-11751-3 PMC8496145

[bibr5-13591053221142348] Huang Y Zhao N (2020) Generalized anxiety disorder, depressive symptoms and sleep quality during COVID-19 outbreak in China: A web-based cross-sectional survey. Psychiatry Research 288: 112954. 32325383 10.1016/j.psychres.2020.112954 PMC7152913

[bibr6-13591053221142348] Iosifyan M Arina G (2021) Perceived value threats are related to fear of health impairments. The Journal of Social Psychology. Epub ahead of print 26 October 2021. DOI: 10.1080/00224545.2021.1979453. 34697996

[bibr7-13591053221142348] Lerner JS Keltner D (2001) Fear, anger, and risk. Journal of Personality and Social Psychology 81(1): 146–159. 11474720 10.1037//0022-3514.81.1.146

[bibr8-13591053221142348] Lindeman M Verkasalo M (2005) Measuring values with the short Schwartz’s value survey. Journal of Personality Assessment 85(2): 170–178. 16171417 10.1207/s15327752jpa8502_09

[bibr9-13591053221142348] Mertens G Gerristen L Salemink E , et al. (2020) Fear of the coronavirus (COVID-19): Predictors in an online study conducted in March 2020. Journal of Anxiety Disorders 74: 102258. 32569905 10.1016/j.janxdis.2020.102258 PMC7286280

[bibr10-13591053221142348] Milfont TL Sibley CG Duckitt J (2010) Testing the moderating role of the components of norm activation on the relationship between values and environmental behavior. Journal of Cross-Cultural Psychology 41(1): 124–131.

[bibr11-13591053221142348] Nelissen RMA Dijker AJM de Vries NK (2007) Emotions and goals: Assessing relations between values and emotions. Cognition & Emotion 21(4): 902–911.

[bibr12-13591053221142348] Park CL Russell BS Fendrich M , et al. (2020) Americans’ COVID-19 stress, coping, and adherence to CDC guidelines. Journal of General Internal Medicine 35(8): 2296–2303. 32472486 10.1007/s11606-020-05898-9 PMC7259430

[bibr13-13591053221142348] Pieh C Budimir S Delgadillo J , et al. (2021) Mental health during COVID-19 lockdown in the United Kingdom. Psychosomatic Medicine 83(4): 328–337. 33009276 10.1097/PSY.0000000000000871

[bibr14-13591053221142348] Russo S Roccato M (2009) Values and fear of crime. In Lamont EP (ed.) Social Psychology: New Research. New York, NY: Nova, pp.267–282.

[bibr15-13591053221142348] Schultz PW Gouveia VV Cameron LD , et al. (2005) Values and their relationship to environmental concern and conservation behavior. Journal of Cross-Cultural Psychology 36(4): 457–475.

[bibr16-13591053221142348] Schwartz SH (1992). Universals in the content and structure of values: Theoretical advances and empirical tests in 20 countries. In Zanna M. P. (ed.) Advances in experimental social psychology, vol. 25. San Diego, CA: Academic Press, pp.1–65.

[bibr17-13591053221142348] Schwartz SH (2003) A proposal for measuring value orientations across nations. Questionnaire development report of the European Social Survey. https://www.europeansocialsurvey.org/docs/methodology/core_ess_questionnaire/ESS_core_questionnaire_human_values.pdf

[bibr18-13591053221142348] Schwartz SH (2016) Basic individual values: Sources and consequences. In Sander D Brosch T (eds) Handbook of Value. Oxford: Oxford University Press, pp.63–84.

[bibr19-13591053221142348] Schwartz SH Sagiv L Boehnke K (2000) Worries and values. Journal of Personality 68(2): 309–346. 10820689 10.1111/1467-6494.00099

[bibr20-13591053221142348] Shechner T Britton JC Pérez-Edgar K , et al. (2012) Attention biases, anxiety, and development: Toward or away from threats or rewards? Depression and Anxiety 29(4): 282–294. 22170764 10.1002/da.20914 PMC3489173

[bibr21-13591053221142348] Winter T Riordan BC Pakpour AH , et al. (2020) Evaluation of the English version of the fear of COVID-19 scale and its relationship with behavior change and political beliefs. International Journal of Mental Health and Addiction 21: 372–382. 32837431 10.1007/s11469-020-00342-9 PMC7295324

[bibr22-13591053221142348] Wolf LJ Haddock G Manstead ASR , et al. (2020) The importance of (shared) human values for containing the COVID-19 pandemic. British Journal of Social Psychology 59(3): 618–627. 32572981 10.1111/bjso.12401 PMC7361394

