# Supplementary material for: Beliefs about COVID-19 as a threat to values are related to preventive behaviors and fear of COVID-19
Source: J Health Psychol. 2023 Jan 2;28(8):739–46. doi: 10.1177/13591053221142348 (PMC9810504; doi:10.1177/13591053221142348)
Supplement: sj-pdf-1-hpq-10.1177_13591053221142348 – Supplemental material for Beliefs about COVID-19 as a threat to values are related to preventive behaviors and fear of COVID-19 [file sj-pdf-1-hpq-10.1177_13591053221142348.pdf]

File	Description
Title Page.docx	Title page with author information, ethics statement, data access statement and conflict of interest statement
Brief report.docx	Main documents without author details
Supplementary materials.docx	Supplementary materials
Table1.pdf	Table
Research Data.rar	Research data including row data, data output, data syntax and an explanatory memo

This research describes two studies investigating links between values (independent variables) and fear of Covid-19 and preventive behaviors (dependent variables).
